# Supplementary material for: Modeling glioblastoma heterogeneity as a dynamic network of cell states
Source: Mol Syst Biol. 2021 Sep 16;17(9):e10105. doi: 10.15252/msb.202010105 (PMC8444284; doi:10.15252/msb.202010105)
Supplement: Supplementary file 6 — Source Data for Figure 5 [file MSB-17-e10105-s004.zip › Figure5A_sourcedata/GSEA_3071/nosigdb_stateIV.GseaPreranked.1622795164626/IVY_CTMVP.html]

Details for gene set IVY\_CTMVP[GSEA]

|  || Dataset | state53071 |
| Phenotype | NoPhenotypeAvailable |
| Upregulated in class | na\_neg |
| GeneSet | IVY\_CTMVP |
| Enrichment Score (ES) | -0.67925787 |
| Normalized Enrichment Score (NES) | -3.5224836 |
| Nominal p-value | 0.0 |
| FDR q-value | 0.0 |
| FWER p-Value | 0.0 |
Table: GSEA Results Summary

  

Fig 1: Enrichment plot: IVY\_CTMVP      
 Profile of the Running ES Score & Positions of GeneSet Members on the Rank Ordered List

  

| PROBE | GENE SYMBOL | GENE\_TITLE | RANK IN GENE LIST | RANK METRIC SCORE | RUNNING ES | CORE ENRICHMENT || 1 | PDGFB |  |  | 86 | 1.615 | -0.0017 | No |
| 2 | CCDC102B |  |  | 93 | 1.559 | 0.0115 | No |
| 3 | STC1 |  |  | 97 | 1.547 | 0.0251 | No |
| 4 | COL1A2 |  |  | 137 | 1.425 | 0.0307 | No |
| 5 | A2M |  |  | 188 | 1.283 | 0.0329 | No |
| 6 | PLXDC1 |  |  | 323 | 1.000 | 0.0163 | No |
| 7 | INPP4B |  |  | 328 | 0.991 | 0.0247 | No |
| 8 | GNG11 |  |  | 490 | 0.823 | 0.0013 | No |
| 9 | MCAM |  |  | 986 | 0.528 | -0.0890 | No |
| 10 | ITGA4 |  |  | 1355 | 0.427 | -0.1558 | No |
| 11 | PTP4A3 |  |  | 1480 | 0.395 | -0.1760 | No |
| 12 | RAMP2 |  |  | 1537 | 0.381 | -0.1832 | No |
| 13 | HAPLN1 |  |  | 1612 | 0.366 | -0.1941 | No |
| 14 | KCNJ2 |  |  | 1722 | 0.346 | -0.2118 | No |
| 15 | CDH6 |  |  | 1920 | 0.310 | -0.2468 | No |
| 16 | KCNJ8 |  |  | 1933 | 0.308 | -0.2463 | No |
| 17 | FRZB |  |  | 2074 | 0.287 | -0.2706 | No |
| 18 | ETS1 |  |  | 2305 | 0.254 | -0.3125 | No |
| 19 | STEAP1B |  |  | 2462 | -0.265 | -0.3400 | No |
| 20 | MECOM |  |  | 2752 | -0.301 | -0.3928 | No |
| 21 | ITGB1 |  |  | 2817 | -0.308 | -0.4022 | No |
| 22 | LAMB1 |  |  | 3029 | -0.336 | -0.4397 | No |
| 23 | ANGPT2 |  |  | 3068 | -0.341 | -0.4439 | No |
| 24 | SVIL |  |  | 3218 | -0.363 | -0.4692 | No |
| 25 | COL18A1 |  |  | 3266 | -0.370 | -0.4748 | No |
| 26 | OLFML2B |  |  | 3411 | -0.392 | -0.4989 | No |
| 27 | MYO1B |  |  | 3431 | -0.396 | -0.4989 | No |
| 28 | PXDN |  |  | 3640 | -0.434 | -0.5349 | No |
| 29 | CRIP1 |  |  | 3651 | -0.435 | -0.5328 | No |
| 30 | PDE5A |  |  | 3672 | -0.438 | -0.5326 | No |
| 31 | FOXF2 |  |  | 3784 | -0.462 | -0.5497 | No |
| 32 | ARHGAP18 |  |  | 3804 | -0.467 | -0.5491 | No |
| 33 | FOXC1 |  |  | 3822 | -0.470 | -0.5480 | No |
| 34 | PLAC9 |  |  | 4137 | -0.551 | -0.6033 | No |
| 35 | ISLR |  |  | 4155 | -0.556 | -0.6015 | No |
| 36 | EDNRA |  |  | 4232 | -0.575 | -0.6108 | No |
| 37 | IGFBP7 |  |  | 4331 | -0.607 | -0.6240 | No |
| 38 | F2R |  |  | 4396 | -0.634 | -0.6305 | No |
| 39 | FSTL1 |  |  | 4446 | -0.653 | -0.6339 | No |
| 40 | PCDH18 |  |  | 4660 | -0.769 | -0.6678 | No |
| 41 | FILIP1L |  |  | 4671 | -0.778 | -0.6626 | No |
| 42 | PHACTR2 |  |  | 4733 | -0.817 | -0.6668 | No |
| 43 | S1PR3 |  |  | 4799 | -0.865 | -0.6713 | Yes |
| 44 | MGP |  |  | 4824 | -0.890 | -0.6677 | Yes |
| 45 | SERPINH1 |  |  | 4829 | -0.895 | -0.6603 | Yes |
| 46 | CTGF |  |  | 4852 | -0.915 | -0.6561 | Yes |
| 47 | EBF1 |  |  | 4856 | -0.919 | -0.6482 | Yes |
| 48 | NR2F2 |  |  | 4870 | -0.937 | -0.6421 | Yes |
| 49 | KDELR3 |  |  | 4912 | -0.981 | -0.6410 | Yes |
| 50 | ITGA1 |  |  | 4951 | -1.019 | -0.6389 | Yes |
| 51 | PAPSS2 |  |  | 4981 | -1.058 | -0.6348 | Yes |
| 52 | PRKG1 |  |  | 4998 | -1.089 | -0.6279 | Yes |
| 53 | UACA |  |  | 5013 | -1.113 | -0.6203 | Yes |
| 54 | LBH |  |  | 5038 | -1.168 | -0.6142 | Yes |
| 55 | PROCR |  |  | 5072 | -1.228 | -0.6093 | Yes |
| 56 | MFAP2 |  |  | 5087 | -1.259 | -0.6004 | Yes |
| 57 | COL5A2 |  |  | 5097 | -1.282 | -0.5904 | Yes |
| 58 | TGFBI |  |  | 5104 | -1.305 | -0.5795 | Yes |
| 59 | PDLIM1 |  |  | 5142 | -1.392 | -0.5738 | Yes |
| 60 | OLFML2A |  |  | 5147 | -1.410 | -0.5616 | Yes |
| 61 | BGN |  |  | 5160 | -1.486 | -0.5503 | Yes |
| 62 | PRSS23 |  |  | 5179 | -1.562 | -0.5394 | Yes |
| 63 | GPX8 |  |  | 5184 | -1.588 | -0.5256 | Yes |
| 64 | CD248 |  |  | 5192 | -1.622 | -0.5120 | Yes |
| 65 | IGFBP4 |  |  | 5211 | -1.701 | -0.4999 | Yes |
| 66 | OLFML3 |  |  | 5214 | -1.736 | -0.4843 | Yes |
| 67 | THBS1 |  |  | 5222 | -1.800 | -0.4691 | Yes |
| 68 | TM4SF1 |  |  | 5228 | -1.853 | -0.4530 | Yes |
| 69 | COL4A2 |  |  | 5235 | -1.917 | -0.4366 | Yes |
| 70 | DCN |  |  | 5237 | -1.928 | -0.4191 | Yes |
| 71 | SNAI2 |  |  | 5238 | -1.940 | -0.4012 | Yes |
| 72 | TPM2 |  |  | 5242 | -1.971 | -0.3837 | Yes |
| 73 | PCOLCE |  |  | 5247 | -2.050 | -0.3656 | Yes |
| 74 | PDGFRB |  |  | 5253 | -2.141 | -0.3469 | Yes |
| 75 | CFH |  |  | 5256 | -2.163 | -0.3274 | Yes |
| 76 | CTHRC1 |  |  | 5265 | -2.365 | -0.3072 | Yes |
| 77 | ACTG2 |  |  | 5266 | -2.417 | -0.2850 | Yes |
| 78 | COL4A1 |  |  | 5270 | -2.478 | -0.2629 | Yes |
| 79 | LUM |  |  | 5271 | -2.487 | -0.2400 | Yes |
| 80 | MYL9 |  |  | 5272 | -2.509 | -0.2170 | Yes |
| 81 | ARHGAP29 |  |  | 5279 | -2.693 | -0.1934 | Yes |
| 82 | FN1 |  |  | 5284 | -3.017 | -0.1664 | Yes |
| 83 | TAGLN |  |  | 5285 | -3.021 | -0.1387 | Yes |
| 84 | FOXS1 |  |  | 5286 | -3.055 | -0.1106 | Yes |
| 85 | COL3A1 |  |  | 5287 | -3.508 | -0.0784 | Yes |
| 86 | COL6A3 |  |  | 5288 | -3.654 | -0.0448 | Yes |
| 87 | COL1A1 |  |  | 5290 | -4.897 | -0.0000 | Yes |
Table: GSEA details [plain text format]

  

Fig 2: IVY\_CTMVP: Random ES distribution      
 Gene set null distribution of ES for **IVY\_CTMVP**
